# Sex- and brain region-specific patterns of gene expression associated with socially-mediated puberty in a eusocial mammal

**DOI:** 10.1371/journal.pone.0193417

**Published:** 2018-02-23

**Authors:** Mariela Faykoo-Martinez, D. Ashley Monks, Iva B. Zovkic, Melissa M. Holmes

**Affiliations:** 1 Department of Cell & Systems Biology, University of Toronto, Toronto, ON, Canada; 2 Department of Psychology, University of Toronto Mississauga, Mississauga, ON, Canada; 3 Department of Ecology and Evolutionary Biology, University of Toronto, Toronto, ON, Canada; University of Texas at Austin, UNITED STATES

## Abstract

The social environment can alter pubertal timing through neuroendocrine mechanisms that are not fully understood; it is thought that stress hormones (e.g., glucocorticoids or corticotropin-releasing hormone) influence the hypothalamic-pituitary-gonadal axis to inhibit puberty. Here, we use the eusocial naked mole-rat, a unique species in which social interactions in a colony (i.e. dominance of a breeding female) suppress puberty in subordinate animals. Removing subordinate naked mole-rats from this social context initiates puberty, allowing for experimental control of pubertal timing. The present study quantified gene expression for reproduction- and stress-relevant genes acting upstream of gonadotropin-releasing hormone in brain regions with reproductive and social functions in pre-pubertal, post-pubertal, and opposite sex-paired animals (which are in various stages of pubertal transition). Results indicate sex differences in patterns of neural gene expression. Known functions of genes in brain suggest stress as a key contributing factor in regulating male pubertal delay. Network analysis implicates neurokinin B (*Tac3*) in the arcuate nucleus of the hypothalamus as a key node in this pathway. Results also suggest an unappreciated role for the nucleus accumbens in regulating puberty.

## Introduction

The biological process of puberty marks the onset of reproductive maturity, and is characterized by striking morphological, physiological and neurobiological changes. The timing of pubertal onset varies within and between sexes of diverse species [1–8], having implications for reproductive fitness [9–10]. Variation in pubertal timing also affects health in humans. Data from the past century indicate that puberty is occurring at a younger age [9, 11–12], with early puberty onset linked to increased incidence of negative health outcomes in adolescence (e.g. depression [13]) and adulthood (e.g. breast cancer [14–16]). While mechanisms underlying individual differences in pubertal timing are not yet fully understood, it is known that environmental factors, particularly social cues and interactions, contribute to population variability in timing within a species. For example, in female rhesus macaques *(Macaca mulatta*), peer aggression delays pubertal timing [17], while housing female laboratory guinea pigs (*Cavia aperea*) [18] or mice (*Mus musculus)* [19] with an adult male causes a shift to earlier puberty onset. In humans, environmental changes associated with wartime have been linked with delayed puberty in females [20–21], while other stressors result in an earlier onset in both sexes [22–25].

Puberty is initiated by activation of the hypothalamic-pituitary-gonadal (HPG) axis. At the onset of puberty, a small neuronal population in the pre-optic area releases gonadotropin-releasing hormone (GnRH) [26–28], causing the pituitary gland to release luteinizing and follicle stimulating hormone, ultimately triggering steroid hormone production by the gonads. The GnRH neurons are themselves regulated by a subpopulation of kisspeptin-neurokinin B-dynorphin (KNDy) neurons in the arcuate nucleus that act in feedback control of GnRH secretion in sheep (*Ovies aries)* [29–30] and rats *(Rattus norvegicus)* [31]. KNDy neurons have been directly implicated in influencing fertility and puberty; for example, loss-of-function mutations of neurokinin B ligand and receptor (*Tac3 and Tac3r*) result in infertility in humans [32], rodents [33–34], and teleosts [35]. Kisspeptin (*Kiss1)* acts as a stimulatory player in this network, stimulating release of GnRH via projections to GnRH neurons in the pre-optic area and median eminence [30, 36]. Consequently, antagonists of the kisspeptin receptor (*Kiss1r*) significantly reduce the luteinizing hormone (LH) surge characteristic of the pre-ovulatory phase [37–38]. Conversely, dynorphin inhibits pulsatile GnRH secretion [39] while neurokinin B can both stimulate and inhibit GnRH secretion depending on coincident endocrine signalling [40–41]. GnRH release is also inhibited by RFamide-related peptide-3 (RFRP-3; mammalian ortholog to gonadotrophin inhibitory hormone, GnIH), which might serve as an additional neuroendocrine signal controlling the onset of puberty [42–44].

Glucocorticoids modulate life history transitions (e.g., dispersal) across vertebrates [45] and are a key mechanism via which environmental cues can alter pubertal neuroendocrine signaling. The hypothalamic-pituitary-adrenal (HPA) axis is activated by stressors, with neurons in the paraventricular nucleus of the hypothalamus releasing corticotropin-releasing hormone, causing the pituitary gland to secrete adrenocorticotropic hormone, which in turns stimulates glucocorticoid release from the adrenal cortex. These circulating glucocorticoids then interact with the hippocampus, paraventricular nucleus and the pituitary gland through negative feedback. Administration of the synthetic glucocorticoid dexamethasone to fetal [46] or pre-pubertal [47] female rats delays pubertal onset as measured by vaginal opening. Chronic administration of corticotropin-releasing hormone also delays puberty in female rats [48]. While glucocorticoid exposure in utero can influence GnRH neuron morphology and function [49–51], the effects of stress on pubertal timing may be, at least in part, due to postnatal alterations in KNDy neuron signaling [52]. Pre-pubertal dexamethasone reduces *Kiss1r* mRNA in female rat hypothalamus [47] and corticosterone decreases activation of Kiss1 neurons, ultimately suppressing LH surges, in post-pubertal female mice [53]. The KNDy neuron ligand-receptor pair *Tac3/Tac3r* is required for stress effects on LH pulse frequency: antagonism of Tac3r blocks lipopolysaccharide-induced delays in the LH pulses of adult female rats [54]. Not only does stress influence pubertal timing but pubertal status (i.e. pre-, peri- or post-pubertal) itself affects HPA axis responsiveness to social stressors [55–62], making the interactions between stress and reproductive neuroendocrinology crucial for understanding how the social environment mediates puberty onset.

Naked mole-rats (*Heterocephalus glaber*) are eusocial rodents that undergo socially-mediated pubertal suppression [63]. This pre-pubertal state persists indefinitely throughout their adult life unless they are released from social suppression, making it a unique phenomenon amongst mammals and allowing for experimental control over the initiation of puberty. Naked mole-rats live in large colonies of up to 300 individuals in which reproduction is restricted to a single breeding female and 1–3 male breeders [64–65]. All other animals are non-reproductive subordinates (i.e., pre-pubertal), who, if removed from the suppressive cues of the breeding female, will undergo endocrine and behavioral transitions characteristic of mammalian puberty [66–68]. There is plasticity in brain and body morphology associated with the transition, including the emergence of sex differences that are not present prior to puberty [69–72]. Interestingly, reproductive suppression is more rigid in females than in males [65, 73] suggesting potential sex differences in mechanisms underlying pubertal suppression.

Although timing of puberty differs between naked mole-rats and common laboratory species studied in puberty research like rats or mice, the key components of the pubertal transition (i.e. HPG axis activation) are conserved [70, 74–75]. Prior to puberty, subordinate naked mole-rats have low progesterone, testosterone and LH concentrations; progesterone and testosterone can increase within a week of colony separation, with sexual behavior and sex-typical distribution of steroid hormone receptors in sociosexual neural circuits following soon thereafter [65, 70, 73]. While GnRH cell number does not vary with sex or status, female breeders have more kisspeptin immunoreactive cells in the rostral periventricular region of the third ventricle and anterior periventricular hypothalamic nuclei relative to female subordinates and male breeders [74]. Subordinate naked mole-rats of both sexes have higher RFRP-3 expression than do breeders in the arcuate and paraventricular hypothalamic nuclei (with extensive fibre distribution), while exogenous RFRP-3 suppresses pubertal onset in animals removed from the suppressive cues of the colony [75]. The sex-by-status differences in hypothalamic kisspeptin, but not RFRP-3, suggests sex-specific regulation of reproduction by KNDy neurons; no research has been reported on neurokinin B and dynorphin A in naked mole-rats.

**Fig 1:**
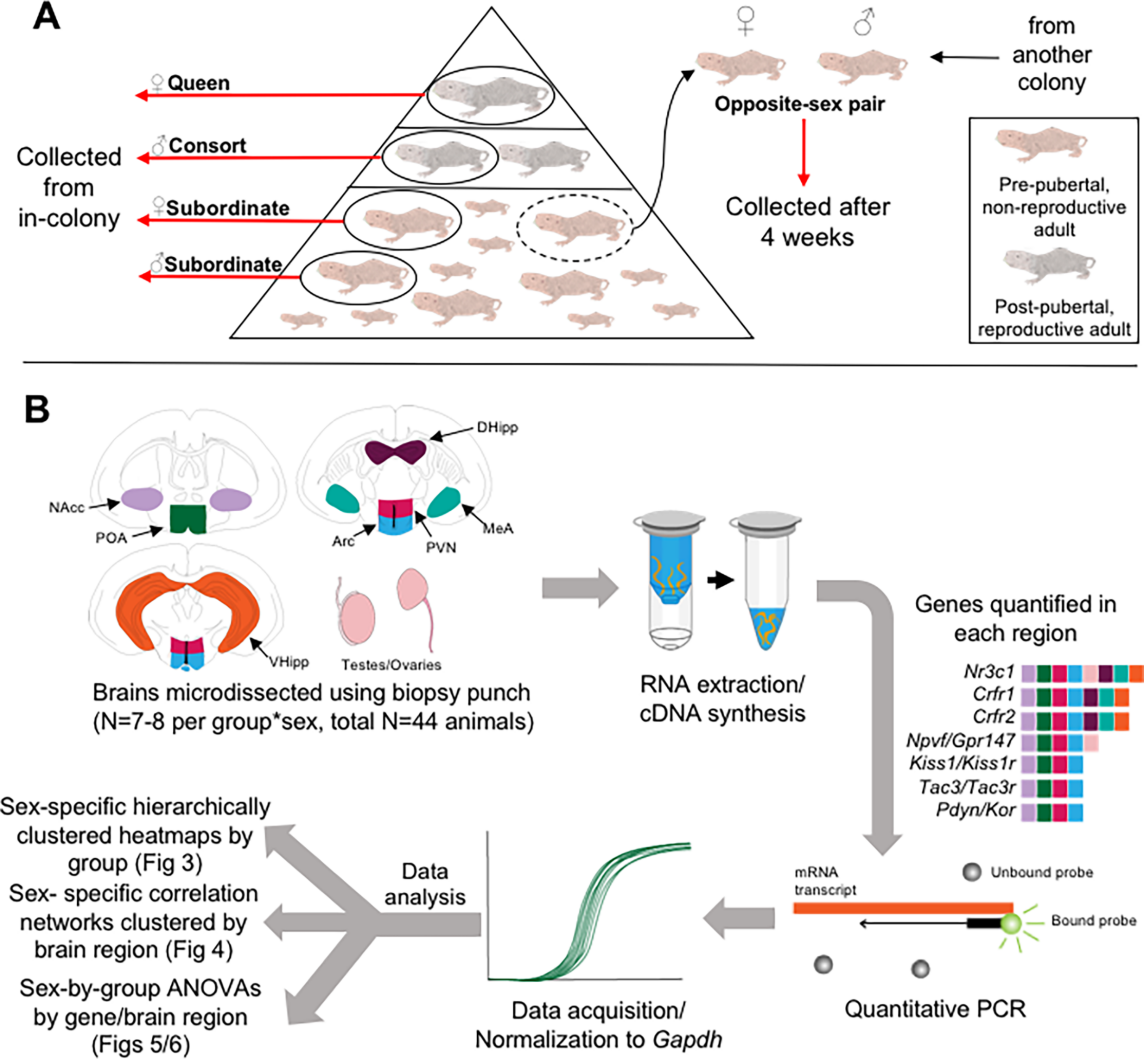
Experimental design and workflow. A) Naked mole-rats live in large colonies with strict reproductive hierarchies. The only reproductively-active animals are the breeding female and 1–3 breeding males; all other animals are reproductively-inactive, pre-pubertal subordinates regardless of age. An animal removed from the suppressive cues of the colony can be paired with an unfamiliar conspecific to trigger pubertal onset. In this study, we collected breeders (N = 14) and subordinates (N = 14) of both sexes from in-colony, in addition to animals that had been paired with an opposite sex animal for four weeks (N = 16). B) 7 brain regions (NAcc = nucleus accumbens, lavender; POA = pre-optic area, green; DHipp = dorsal hippocampus, purple; MeA = medial amygdala, turquoise; PVN = paraventricular nucleus/dorsomedial nucleus; pink; Arc = arcuate nucleus/median eminence, blue; VHipp = ventral hippocampus, orange) and gonads were collected from all animals, followed by RNA extraction and cDNA synthesis. Quantitative PCR was then used to determine relative gene expression, with the brain regions in which a gene was quantified indicated by a square of the same colour (i.e. a gene with a lavender box means it was quantified in the NAcc). Data were normalized to *Gapdh* and analyzed in three ways: sex-specific hierarchically clustered heatmaps (Fig 3), sex-specific correlation networks (Fig 4), and sex-by-group ANOVAs for individual gene analysis (Figs 5 and 6).

**Fig 2:**
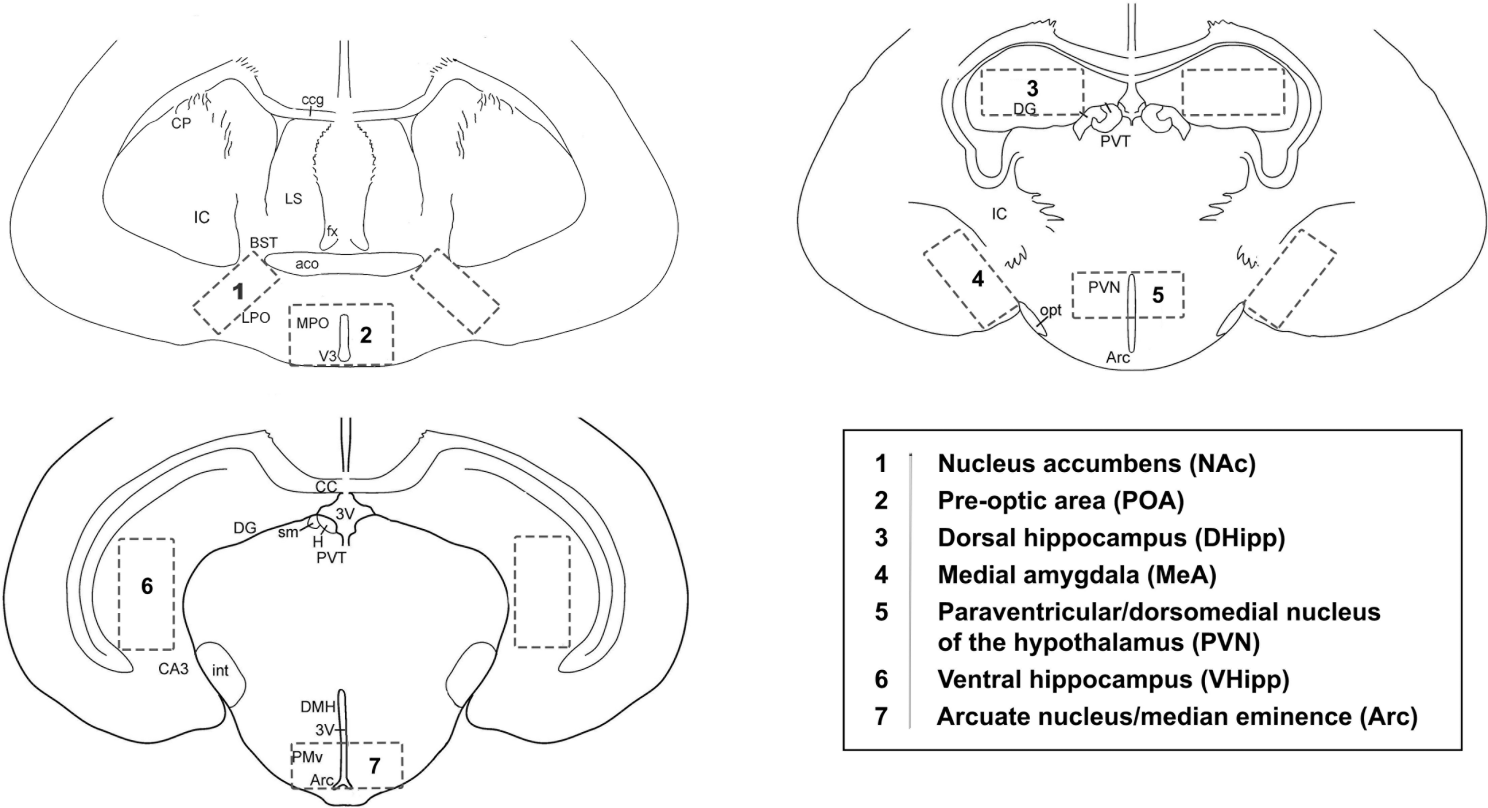
Schematic of representative sections sliced at 2-mm using the brain matrix. A biopsy punch (1-mm diameter) was used to take 4 punches bilaterally (i.e. 2 punches on left, 2 punches on right) in each of the 7 regions depicted.

To identify candidate genes and circuits involved in socially-mediated pubertal suppression in naked mole-rats, we quantified expression of several reproduction- and stress-relevant genes that act upstream of GnRH (outlined in Fig 1). We compared subordinate and breeding animals, in addition to a transition group of reproductively-activated, non-breeding animals (opposite-sex paired; OS; described in [76]) (Fig 1A). OS animals are removed from the suppressive cues of the colony, but while they show evidence of reproductive maturation, they have yet to produce a litter. Their role in this design served to tease apart social suppression from actual reproduction. Specific brain regions were selected for their importance to reproduction and stress (Fig 1B, Fig 2). We also measured circulating cortisol, testosterone and progesterone to measure how stress and reproductive status are associated with gene expression levels in the brain. We hypothesized that gene expression varies in a brain region- and sex-specific manner to co-ordinate the neuroendocrine signalling required for reproductive suppression and subsequent maturation. We predicted genes imperative to maintaining pubertal suppression would be elevated in relevant regions in subordinates, but not in OS or breeding animals. Furthermore, we predicted sex differences in gene expression in subordinate animals due to the higher degree of reproductive suppression evident in subordinate females of this species.

## Methods

### Animals and housing

A total of 44 naked mole-rats from three sex-balanced groups were used: colony-housed breeders (N = 14), colony-housed subordinates (N = 14) and opposite-sex pair-housed subordinates (OS, N = 16). Breeding pairs and sex-matched subordinate animals were taken from 7 colonies, while paired animals were taken from 9 colonies. OS animals were unfamiliar conspecifics, one animal of each sex, paired together in a new cage for 4 weeks. Animals were age-matched across colonies and fell within the following age and weight ranges: subordinates (N = 7 per sex) were 1–9.5 years old and weighed 27–59 g, OS animals (N = 8 per sex) were 0.75–3.5 years old and weighed 32–57 g, and breeders (N = 7 per sex) were 3.5–8 years old and weighed 31–74 g.

All colonies were housed in polycarbonate cages of three sizes (large: 65 cm L x 45 cm W x 23 cm H; medium: 46 cm L x 24 cm W x 15 cm H; small: 30 cm L x 18 cm W x 13 cm H) connected by tubes (25 cm L x 18 cm D) and lined with corn cob bedding. Opposite-sex pairs were housed in a single, medium polycarbonate cage. Naked mole-rats were kept on a 12:12 light/dark cycle at 28–30°C and fed ad libitum with a diet consisting of sweet potato and wet 19% protein mash (Harlan Laboratories Inc.). All experimental procedures followed federal and institutional guidelines and were approved by the University Animal Care Committee.

### Tissue collection

Animals were removed from colony (breeders and subordinates) or pair housing (OS) and immediately anaesthetized in an isoflurane chamber. They were then quickly weighed and decapitated, and trunk blood was collected. All animals were decapitated within 5 minutes of handling the cage. Blood samples were kept on wet ice until centrifugation, and serum stored at -20°C. Brains and gonads were extracted from animals, frozen in liquid nitrogen and stored at -80°C until sub-dissections. At a later date, brains were sliced at 2-mm intervals in a mouse brain matrix on dry ice in a sterile environment. An Integra Miltex biopsy punch (Cat. No. 33-31AA-P/25) was used to extract four tissue punches (1-mm diameter; bilateral; 2 per hemisphere) from each of the following brain regions: pre-optic area; nucleus accumbens; medial amygdala; paraventricular/dorsomedial nuclei of the hypothalamus; dorsal hippocampus; ventral hippocampus; and arcuate nucleus/median eminence (Figs 1 and 2). Both sides of the sectioned tissue were checked while punching to assess for regional overlap. Dissected regions were then stored at -80°C in sterile tubes until RNA extraction.

### Sample preparation and quantitative Polymerase Chain Reaction (qPCR)

RNA was extracted from all samples (brain regions and gonads) using a BioBasic kit (Cat. No. BS82322) and treated with DNase I (Qiagen, Cat. No. 79254). Sample concentration and quality was determined using a Nanodrop 1000 spectrophotometer. cDNA was then synthesized using ThermoFisher Scientific reverse transcriptase (Cat. No. 4368814) and diluted 1:5 to 2 ng/μL. A summary of genes selected and their function can be found in Table 1. Species-specific primer sequences used for performing qPCR were optimized through a standard dilution and melt curve, listed in Table 2. qPCR was run using ABM Evagreen master mix (Cat. No. MasterMix-R) on a BioRad CFX Connect^TM^ (Cat. No. 1855201) with a 10 min incubation at 95°C followed by 40 cycles (15 seconds denature, 60 seconds annealing). Each 10 μL reaction contained 2 μL of cDNA and was run in triplicate. 3% dimethyl sulfoxide was added to the reaction mixture for *Kiss1* due to high G/C content and number of repeats in the primer sequence. For each gene, individual animals and brain regions were distributed randomly across different plates with subordinate animals acting as baseline for relative gene expression calculations. For each experimental gene, the triplicate was averaged, normalized first to the *Gapdh* Ct in the same brain region for that individual and then to the average of the control group, subordinates of both sexes (delta delta Ct).

**Table 1:** A summary of genes quantified and their function in neuroendocrine signaling.

Gene	Name	Function
*Crhr1 (Crfr1)*	Corticotropin-releasing hormone receptor 1	Receptor for corticotropin-releasing hormone, a major regulator of the hypothalamic-pituitary-adrenal axis
*Crhr2 (Crfr2)*	Corticotropin-releasing hormone receptor 2	Receptor for corticotropin-releasing hormone, a major regulator of the hypothalamic-pituitary-adrenal axis
*Gpr147 (Npfr1)*	G-protein coupled receptor 147 (RFRP receptor)	Receptor for RFamide related peptide 3 (i.e. ligand produced from *Npvf* following post-translational modifications)
*Kor*	Kappa opioid receptor	Receptor for dynorphin A (i.e. ligand produced from *Pdyn* following post-translational modifications)
*Kiss1*	Kisspeptin	Initiates gonadotropin releasing hormone (GnRH) secretion at puberty and ovulation; co-localizes with dynorphin A and neurokinin B in KNDy neurons for feedback regulation of GnRH neurons
*Kiss1r*	Kisspeptin receptor	Receptor for kisspeptin
*Npvf*	Neuropeptide VF precursor	Precursor for RFamide-related peptide-3, which inhibits GnRH
*Nr3c1 (Gr)*	Glucocorticoid receptor	Receptor for glucocorticoid, involved in negative feedback regulation of hypothalamic-pituitary-adrenal axis
*Pdyn*	Prodynorphin	Precursor for dynorphin A; co-localizes with kisspeptin and neurokinin B in KNDy neurons for feedback regulation of GnRH neurons
*Tac3 (Nk3)*	Tachykinin 3 (Neurokinin B)	Initiates GnRH secretion at puberty and ovulation; co-localizes with kisspeptin and dynorphin A in KNDy neurons for feedback regulation of GnRH neurons
*Tac3r (Nk3r)*	Tachykinin 3 receptor (Neurokinin B receptor)	Receptor for neurokinin B

**Table 2:** Sequences of the primers used.

Gene	Forward Sequence	Reverse Sequence
*Crhr1 (Crfr1)*	GAATCCTTCCAGGTCCGCTC	AGATCTCAGGGGCTGCTTTG
*Crhr2 (Crfr2)*	CATGTGTCTGCTCTGTCTGCT	GCAGTAGGAGTAGGGACCTGAG
*Gap*dh	CCAAGGTCATCCACGACAAT	ACGCTGGGATGATGTTCTG
*Gpr147 (Npffr1)*	ACAACCTCATCACTGGGTGG	GCAGGGTTAGCTTCTCTCGG
*Kor*	CGGATGACGACTACTCCTGG	GTGATCCTGCGGAGGTTTCG
*Kiss1*	ATGAACTCACTGGTTTCCTGG	GATTCTCCACAGGTGCCACCTT
*Kiss1r*	CCAACGGCTCGGATGGC	CCGCCAGGTTAGCGATATAGA
Npvf*	GTTCCAAGCCTAGAGGAACCC	CCCGAATCTCAGTGGCAAGT
*Nr3c1 (Gr)*	CAGGACCACCTCCCAAACTC	TGCTGTCTACCTTCCACTGC
Pdyn*	AAAAGTGGACTCCTCTCCAG	CGTAGCGTTTGACCTGCTCC
*Tac3 (Nk3)*	GCTGAAAGTGCTGAGCAAGG	AGGTGTGTCTGGAAGGCTGT
*Tac3r (Nk3r)*	AAGGCCAAGCGAAAGGTTGT	TGGGGTTGTACATGGTCGAG

*Npvf and Pdyn are precursor peptides for RFRP-3 and Dynorphin A, respectively

### Hormone assays

Progesterone was measured using an enzyme-linked immunosorbent assay (ELISA) kit from Cayman Chemical (Cat. No. 582601) with serum diluted 1:10 in buffer. The assay is sensitive to a minimum of 10 pg/mL with an intra-assay coefficient of variation of less than 12%. There is a manufacturer reported assay cross-reactivity with 17β-estradiol (7.2%), 5β-pregnan-3α-ol-20-one (6.7%), pregnenolone (2.5%), and less than 0.5% with any other hormone or metabolite.

Total testosterone was measured using an ELISA kit from Enzo Life Sciences (Cat. No. ADI-901-065) with serum diluted 1:5 in buffer. The assay is sensitive to a minimum of 5.67 pg/mL with an intra-assay coefficient of variation of 10%. There is a manufacturer reported assay cross-reactivity with 19-hydroxytestosterone (14.6%), androstenedione (7.20%) and less than 1% of other hormones and metabolites.

Cortisol was measured using an ELISA kit from Cayman Chemical (Cat. No. 500360) with serum diluted 1:10 in buffer. The assay is sensitive to a minimum of 80 pg/mL with an intra-assay coefficient of variation of less than 14%. There is a manufacturer reported assay cross-reactivity with dexamethasone (15%), prednisolone (4%), cortexolone (1.6%), and less than 1.0% with any other hormone or metabolite.

A Synergy-HT-Bio-Tek plate reader was used to measure the ELISA plate absorbance at 405 nm for testosterone, progesterone and cortisol. All samples were run in duplicate and the average reported. All protocols were followed as per manufacturer specifications.

### Statistical analyses

All statistical analyses were performed based on the relative enrichment determined for each sample. Enrichment was relative to all subordinates (males and females combined) allowing us to statistically examine sex differences. If enrichment was calculated in a sex-stratified manner (i.e., relative to same sex subordinates), we would not be able to statistically compare expression in males and females. The relative enrichment was calculated as 2^-x^, where x is delta delta Ct. Major outliers were removed prior to analysis, defined as 2 standard deviations above or below the mean of its group and sex. While we attempted to balance colony of origin across experimental groups, we were not always able to do so for the OS group. Thus, animals were statistically treated as independent individuals, regardless of colony origin or pairing as in the case of OS animals. Given the exploratory nature of this study, where our goal was to identify candidate genes and circuits involved in socially-mediated pubertal suppression, we employed a liberal statistical approach.

**Fig 3:**
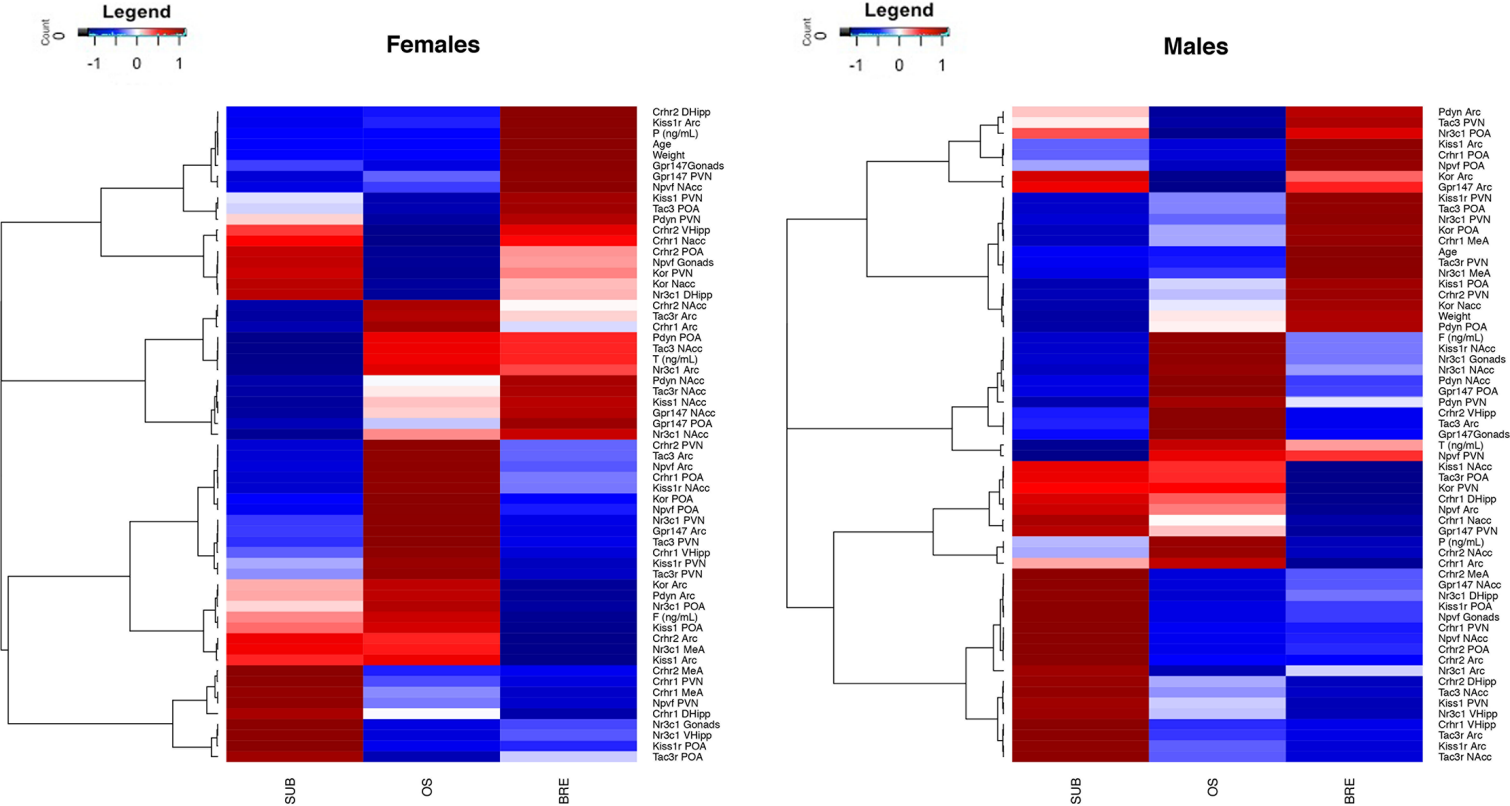
Sex- and status-specific heatmaps. Heatmap of gene expression means for a group in a given brain region, arranged using hierarchical clustering separated by sex (females on left, males on right). Groups are ordered to represent temporal progression through puberty, with SUB (reproductively inactive, socially subordinate) on the left, OS (reproductively active, removed from colony hierarchy) in the middle and BRE (reproductively active, socially dominant) on the right, to visualize how a gene’s expression pattern changes across the transition. Means were adjusted to z-scores with blue indicating low relative expression and red indicating high relative expression. *BRE = breeder*, *SUB = subordinate*, *OS = opposite sex paired*.

First, because neuroendocrine signaling is sexually differentiated in other mammals, gene expression means for each sex were separated by group and plotted on a heatmap to observe sex-specific gene expression patterns between the reproductive groups (Fig 3). The heatmaps were produced in R using the gplots package for plotting, while the stats package was used to produce a hierarchical clustering of genes that associated by similarity in gene expression pattern across the three groups [77]. A scale was used to transform means into z-score values to account for the distribution of values.

**Fig 4:**
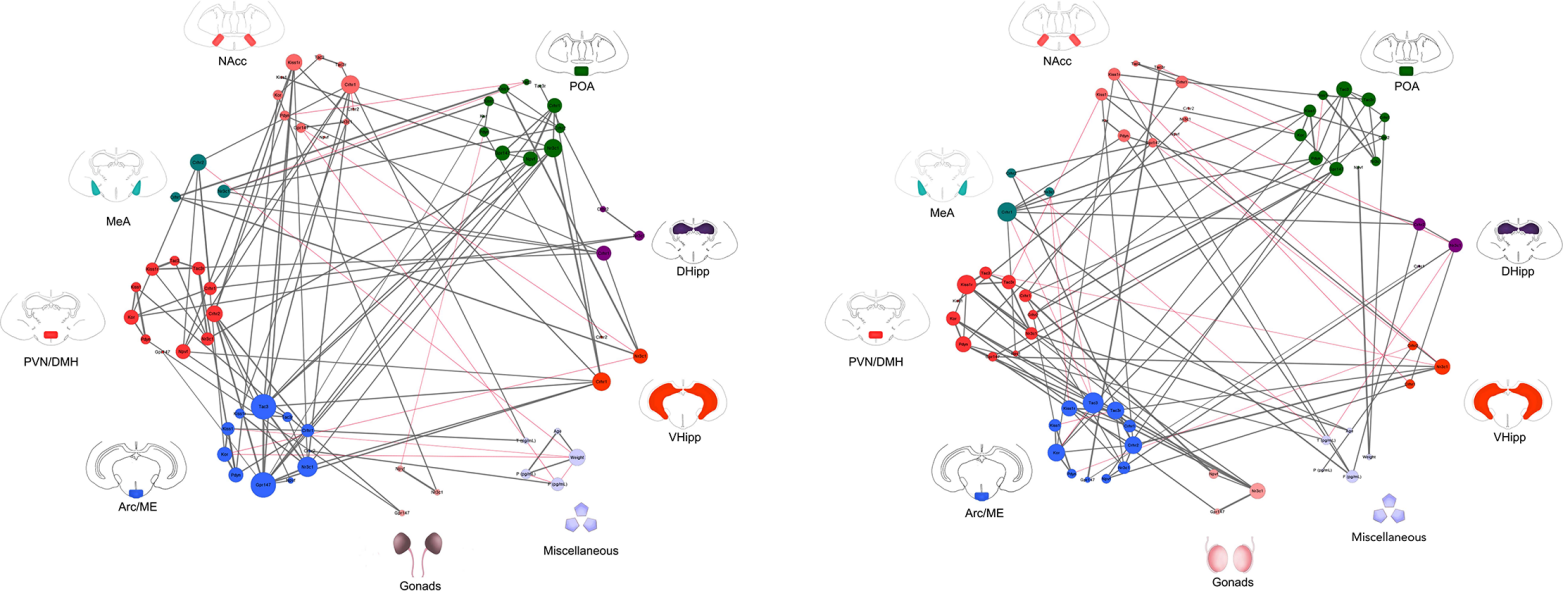
**Correlation networks for females (left) and males (right).** Pair-wise Pearson’s correlations were calculated for relative expression data with a threshold placed at p<0.05 and r>0.4. Genes were clustered and color-coordinated based on brain region. Edge-weight value is visualized through edge thickness (directly correlated to magnitude of the correlation value, r) while node size is a measure of degree (how many edges are connected to a given node). Grey lines denote positive correlations and red lines denote negative correlations. The miscellaneous group encompasses measurements that are not gene expression (hormone data, age, weight) but interact with these data. A description of mapped genes (*Nr3c1*, *Crhr1*, *Crhr2*, *Npvf*, *Gpr147*, *Kiss1*, *Kiss1r*, *Tac3*, *Tac3r*, *Pdyn* and *Kor)* can be found in Table 1. *NAcc = nucleus accumbens*, *POA = pre-optic area*, *Amyg = amygdala*, *PVN = paraventricular/dorsomedial nuclei*, *Arc = arcuate nucleus/median eminence*, *DHipp = dorsal hippocampus*, *VHipp = ventral hippocampus*.

Second, to further explore the clustering of genes as presented by the hierarchical clustering in the heatmaps, sex-specific regional cluster networks were produced (Fig 4). The Hmisc package in R was used to create network edges based on the pair-wise Pearson correlations of all females and all males, with a liberal threshold placed at p<0.05 [78]. Edge data was imported into Cytoscape and genes clustered based on brain region [79]. The Network Analyzer plug-in was used to adjust edge-weight based on correlation strength and node size based on within-network degree (i.e. number of edges for a given node) [80]. Positive correlations were assigned a grey line and negative correlations assigned a red line.

**Fig 5:**
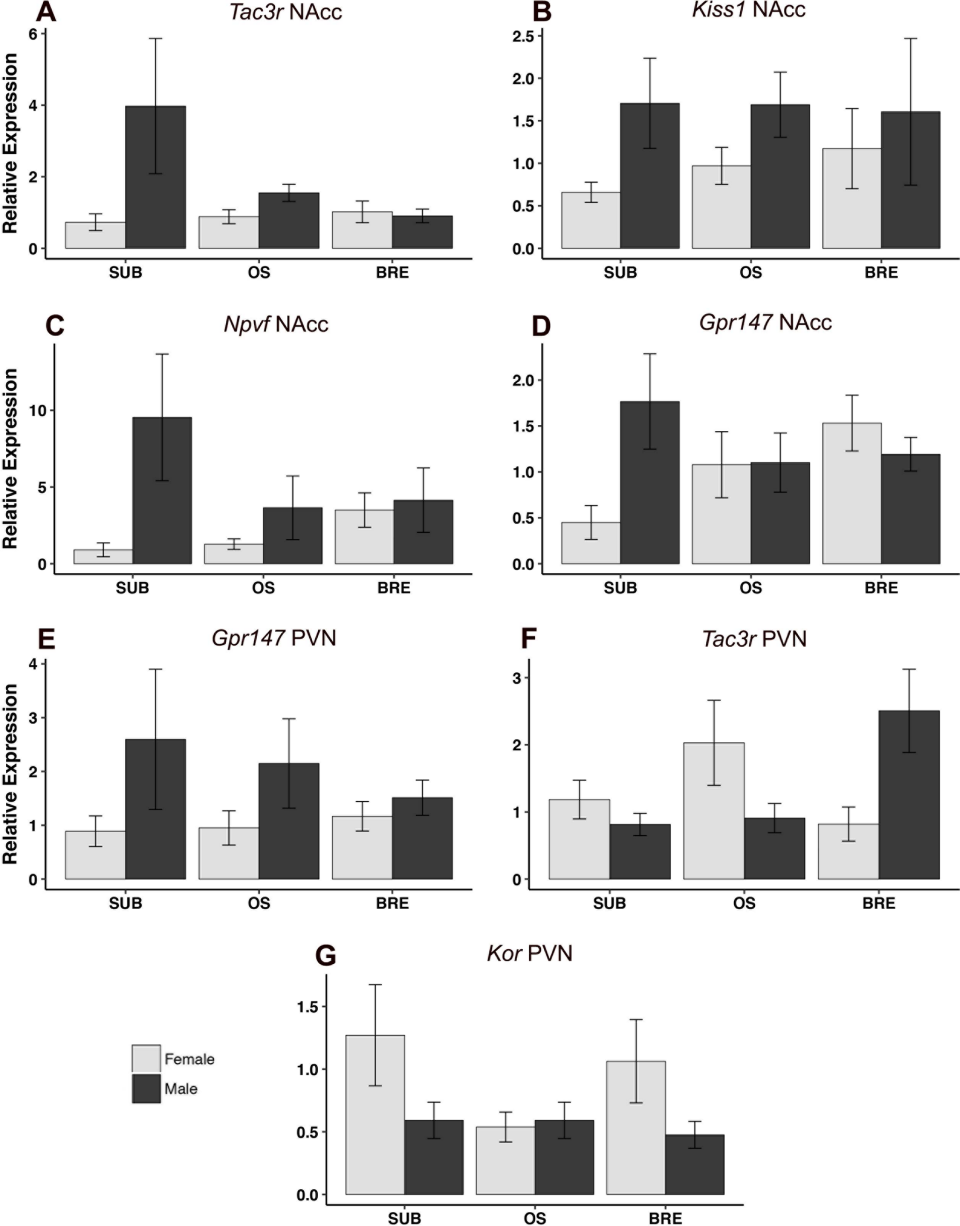
Relative mRNA expression in the NAcc and PVN +/- standard error of the mean. A) *Tac3r*, NAcc: Males had elevated expression as compared to females, p = 0.050. B) *Kiss1*, NAcc: The main effect of sex, favoring males, approached significance, p = 0.052. C) *Npvf*, NAcc: Males had elevated expression as compared to females, p = 0.032. D) *Gpr147*, NAcc: A significant sex-by-group interaction (p = 0.046) was detected. E) *Gpr147*, PVN: Males had elevated expression as compared to females: p = 0.048. F) *Tac3r*, PVN: A significant sex-by-group interaction (p = 0.005) was detected. G) *Kor*, PVN: The main effect of sex, favoring males, approached significance, p = 0.054. No statistically significant post hoc comparisons were detected following the significant main or interaction effects. *NAcc = nucleus accumbens*, *PVN = paraventricular/dorsomedial nucleus*, *BRE = breeder*, *SUB = subordinate*, *OS = opposite sex paired*.

**Fig 6:**
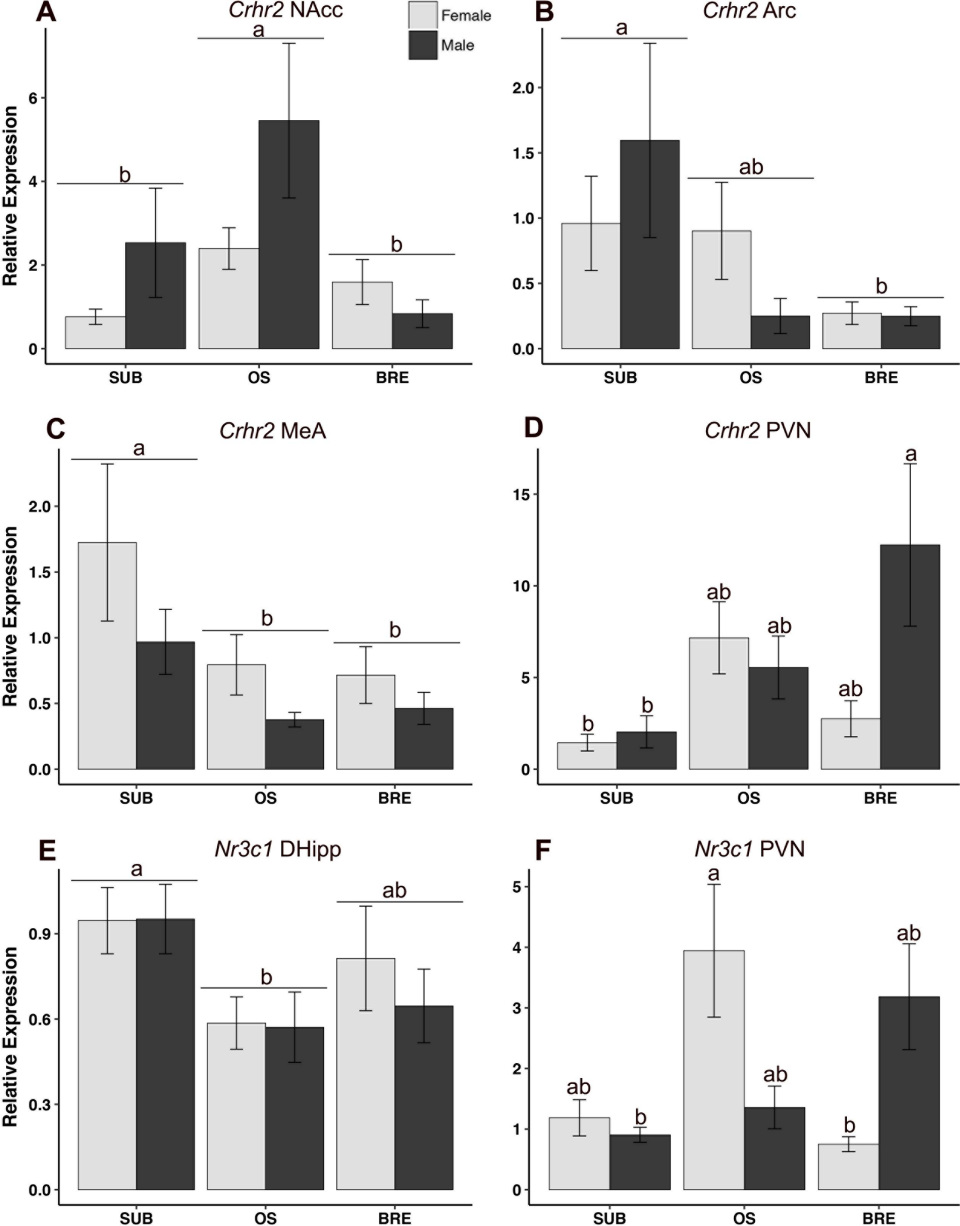
Relative mRNA expression of stress-related genes +/- standard error of the mean. A) *Crhr2*, NAcc: OS animals had highest expression, p = 0.035, irrespective of sex. B) *Crhr2*, Arc: SUB animals had higher expression than BRE, irrespective of sex, p = 0.040. C) *Crhr2*, MeA: SUB animals had higher expression than all other groups, p = 0.023. D) *Crhr2*, PVN: Significant sex-by-group interaction (p = 0.042) revealed BRE males had higher expression relative to SUB females (p = 0.017) and SUB males (p = 0.027) and a trend for higher expression than BRE females (p = 0.06). E) *Nr3c1*, DHipp: SUB animals had higher expression than OS, p = 0.026. F) *Nr3c1*, PVN: Significant sex-by-group interaction (p = 0.002) revealed OS females had higher expression relative to SUB males (p = 0.033) and BRE females (p = 0.023), and a trend for higher expression relative to SUB females (p = 0.067) and OS males (p = 0.062). (a) signifies statistical difference (p<0.05) from (b). *NAcc = nucleus accumbens*, *Arc = arcuate nucleus/median eminence; MeA = medial amygdala; PVN = paraventricular/dorsomedial nucleus*, *VHipp = ventral hippocampus*, *DHipp = dorsal hippocampus*, *BRE = breeder*, *SUB = subordinate*, *OS = opposite sex paired*.

**Fig 7:**
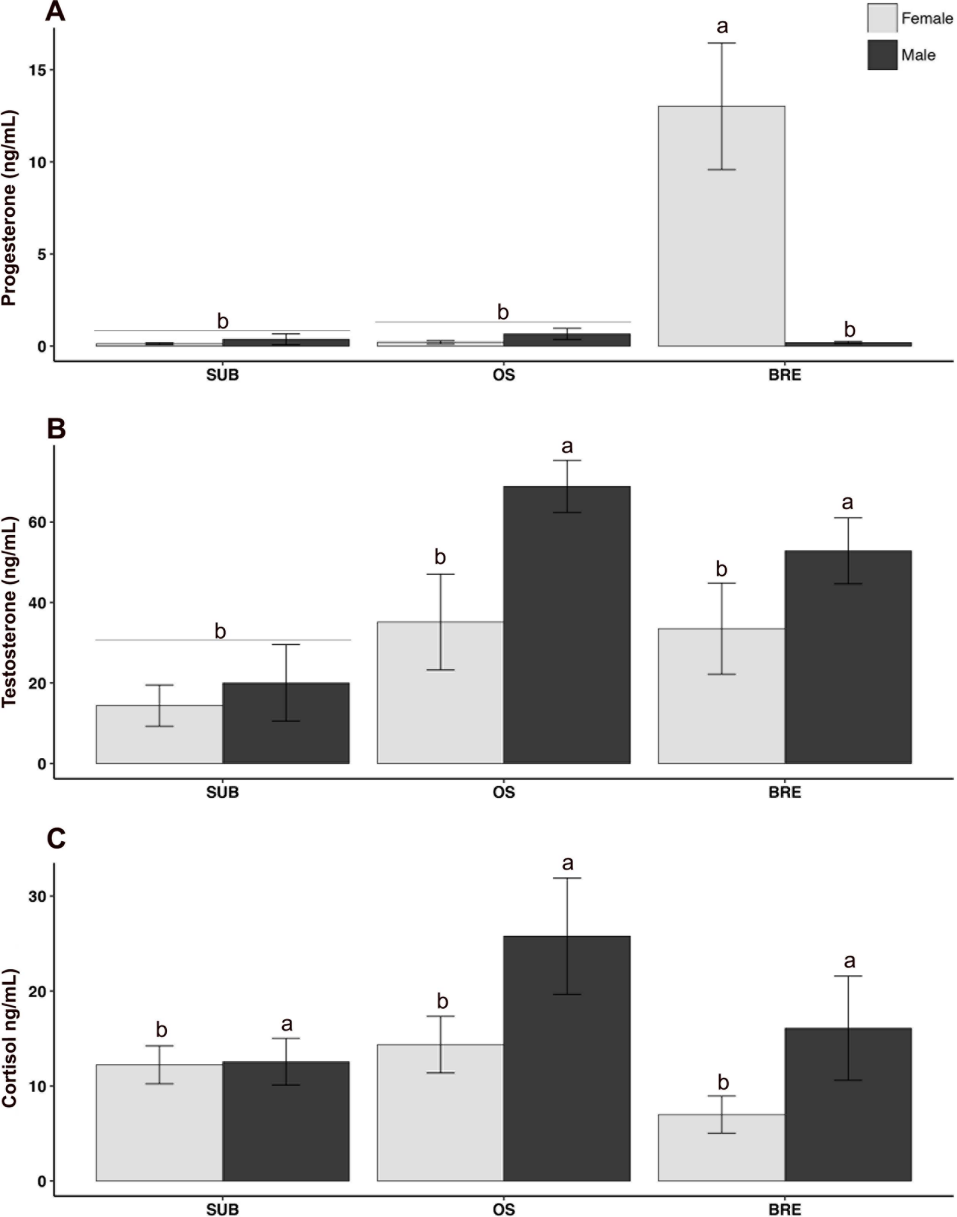
Circulating levels of hormones reported as ng/mL +/- SEM. A) **Progesterone** was significantly elevated in females (p = 0.001) and BRE (p<0.0001), with this effect driven by breeding females specifically (p = 0.001). B) **Testosterone** was significantly elevated in males (p = 0.01) overall, but this was driven by significant elevation in BRE and OS, but not SUB males (p = 0.001). C) **Cortisol** was significantly elevated in males (p = 0.048), with OS animals approaching a statistically significant elevation in circulating cortisol (p = 0.066). (a) signifies statistical difference (p<0.05) from (b). *BRE = breeder*, *SUB = subordinate*, *OS = opposite sex paired*.

Finally, group-by-sex ANOVAs were used to analyze data for relative gene expression in each region, as well as for age, weight, and circulating hormone levels, using the psych package in R, followed by Tukey’s HSD [81]. Results with a p<0.05 were considered statistically significant. ANOVAs were plotted using the ggplot2 package in R (Figs 5–7) [82].

## Results

### Body weight varied by sex and group; age varied by group

Breeders were significantly older than other groups (main effect of group: F(2,38) = 35.0; p<0.0001) though no significant effect of sex (F(1,38) = 0.256; p = 0.616) or interaction (F(2,38) = 0.012; p = 0.988) was detected. A significant main effect of sex (F(1,38) = 8.24; p = 0.007) and group (F(2,38) = 3.83; p = 0.031) was revealed for weight, where females and breeders were heavier, respectively. No significant sex-by-group interaction on weight was detected (F(2,38) = 1.33, p = 0.275).

### Heatmaps and networks reveal sex-specific patterns in gene expression

The heatmaps present a time lapse, using a cross-sectional design, of how gene expression changes from subordinate to OS to breeder in each brain region. In contrast, the networks reveal sex-specific patterns of inter- and intra-regional correlations for gene expression. The heatmaps (Fig 3) and networks (Fig 4) were analyzed visually in parallel to identify patterns; underlying statistical analyses for Figs 3 and 4 use orthogonal methodology. Expected patterns emerge in both female visuals: positive correlations between age, weight, and progesterone. This pattern is expected as female breeders are older, heavier and have significantly higher progesterone (see ANOVA results below) [64, 74, 83].

Several patterns of relative gene expression are observed in both sexes. First, *Nr3c1*, *Kiss1r*, *Tac3* and *Tac3r* form a sub-network in the paraventricular/dorsomedial nuclei. Second, *Kiss1*, *Pdyn* and *Kor* are all highly correlated in the arcuate nucleus. Finally, in both sexes, but more so in females, *Npvf* and *Nr3c1* are highly correlated in the gonads; this could be a subordinate-driven effect given that *Nr3c1* could be acting to repress reproduction by increasing *Npvf*. In contrast, notable sex differences were detected when comparing the relative degree (i.e., number of edges corresponding to the node) of network nodes. The nodes with the highest degree in females are *Tac3*, *Gpr147* and *Nr3c1* in the arcuate nucleus, *Crhr1* in the nucleus accumbens and ventral hippocampus, and *Nr3c1* in the pre-optic area. Furthermore, females have increased intraregional correlations in the arcuate nucleus and increased interregional correlations between the pre-optic area to arcuate nucleus and the paraventricular nucleus to nucleus accumbens. For males, the nodes with the highest degree are *Tac3*, *Tac3r*, *Crhr2* and *Kor* in the arcuate nucleus, *Crhr1* in the medial amygdala and *Kiss1r* in the paraventricular nucleus. Males have several other sex-specific gene expression patterns: 1) *Crhr1* in the paraventricular nucleus is highly correlated to *Npvf* in the gonads; 2) *Tac3*, *Tac3r* and *Crhr1* are highly correlated to one another in the arcuate nucleus; 3) *Gpr147* in the pre-optic area is correlated to *Npvf* in the paraventricular nucleus; 4) *Nr3c1*, *Tac3*, *Tac3r*, *Kiss1*, *Kiss1r* and *Pdyn* are all upregulated in a positive correlation network in the pre-optic area; and 5) *Nr3c1* in the gonads is also correlated to several genes in the paraventricular nucleus and the arcuate nucleus.

### Individual gene analysis reveals sex- and/or group-differences in gene expression

Only results that are statistically significant (p<0.05), or statistical trends (p<0.10), are reported here; all other ANOVA results can be found in Supplementary Table 1.

Expression of genes was influenced by sex in the nucleus accumbens and paraventricular nucleus. Males, regardless of group, had higher expression of *Tac3r* in the nucleus accumbens (main effect of sex: F(1,37) = 4.10, p = 0.05) (Fig 5A), while the main effect of sex on *Kiss1* in this region approached significance (F(1,36) = 4.04, p = 0.052) (Fig 5B). Also in the nucleus accumbens, males had higher expression of *Npvf* (main effect of sex: F(1,36) = 4.96, p = 0.032) (Fig 5C). A significant sex-by-group interaction for *Gpr147* in the nucleus accumbens (F(2,34) = 3.37, p = 0.046) was detected; post-hoc tests were not significant though subordinate males trend towards higher expression than subordinate females (p = 0.100). Similarly, males had higher expression of *Gpr147* in the paraventricular nucleus (F(1,32) = 4.24, p = 0.048) (Fig 5D). A significant sex-by-group interaction was found for *Tac3r* in this region (F(2,37) = 5.99, p = 0.005) (Fig 5E). Post-hoc analyses only approached significance with breeding males having higher expression than breeding females (p = 0.083), subordinate males (p = 0.095) and OS males (p = 0.095). *Kor* in the paraventricular nucleus approached statistical significance where females trended towards higher expression (F(1,33) = 4.00, p = 0.054) (Fig 5F).

Expression of stress-related genes varied according to sex and/or group, depending on brain region. *Crhr*2 in the nucleus accumbens was highest in OS animals (main effect of group: F(2,36) = 3.68, p = 0.035) (Fig 6A) whereas *Crhr2* in the arcuate nucleus (main effect of group: F = (2,34) = 3.54, p = 0.040) was higher in subordinates relative to breeders (Fig 6B) and higher relative to all groups in the medial amygdala (F(2,36) = 4.20, p = 0.023) (Fig 6C). For the paraventricular nucleus, a significant sex-by-group interaction (F(2,37) = 3.45, p = 0.042) and main effect of group (F(2,37) = 3.97, p = 0.027) (Fig 6D) for *Crhr2* reveals breeding males had higher expression than subordinate males (p = 0.027) and subordinate females (p = 0.017), and trended towards higher expression than breeding females (p = 0.063), while subordinates had lower expression overall. A main effect of group was found for the glucocorticoid receptor gene, *Nr3c1*, expression in the dorsal hippocampus (F(2,37) = 4.02, p = 0.026) (Fig 6E) where subordinate animals had higher expression than OS animals. Finally, *Nr3c1* expression also varied according to sex and group in the paraventricular nucleus (F = (2,35) = 7.18, p = 0.002) where OS females had higher expression than breeding females (p = 0.023) and subordinate males (p = 0.033), and trended towards higher expression in OS males (p = 0.062) and subordinate females (p = 0.067) (Fig 6F).

### Circulating steroids varied by sex and/ or group

Significant main effects of sex (F(1,38) = 12.2, p = 0.001) and group (F(2,38) = 14.0, p<0.0001) were observed for circulating progesterone with females and breeders having higher levels, respectively (Fig 7A). A significant group-by-sex interaction effect (F(2,38) = 15.5, p<0.0001) indicated that both significant main effects were driven by breeding females having greatly elevated progesterone (p<0.001 compared to all other groups).

A group-by-sex ANOVA on circulating testosterone revealed a significant main effect of sex (F(1,38) = 7.30, p = 0.01), with males having higher testosterone than females (Fig 7B). A significant main effect of group revealed (F(2,38) = 7.80, p = 0.001) that breeders and OS animals had greater total testosterone than subordinates, reflecting reproductive maturation in these groups. The group-by-sex interaction did not reach statistical significance (F(2,38) = 1.19, p = 0.31).

Overall, males had significantly higher circulating cortisol (main effect of sex: F(1,38) = 4.81, p = 0.048) compared to females (Fig 7C). The main effect of group approached significance (F(2,38) = 2.88, p = 0.066) with OS animals having higher cortisol, while the group-by-sex interaction was non-significant (F(2,38) = 1.07, p = 0.230).

## Discussion

Utilizing expression studies of genes acting upstream of GnRH in several brain regions, we identify neuroendocrine candidates involved in the socially-mediated pubertal suppression seen in naked mole-rats. We employed a liberal, exploratory approach to generate a large amount of data which can serve as the foundation for future directed hypothesis testing. In addition to revealing an under-appreciated association of the nucleus accumbens with this reproductive transition, we identify sex-specific gene expression patterns associated with pubertal transition in a species with reduced sex differences in the brain and behavior [69–70, 74–76, 84–89]. For example, while we have identified neurokinin B (*Tac3*) in the arcuate nucleus as a key node in both males and females, a sub-network of *Tac3/Tac3r/Crhr1* signalling in the arcuate nucleus seems to be exclusive to males, suggesting sex-specific mechanisms for crosstalk between the HPA and HPG axes.

### Sex differences in gene expression are associated with pubertal suppression

The onset of puberty in naked mole-rats manifests in sex-specific ways, as in other mammals. Here, we report that in OS and breeding animals, males had increased circulating testosterone, while female breeders had higher progesterone relative to all other groups [consistent with 56, 62, 65, 68]. Gene expression patterns also differed between sexes, with unique sets of genes interacting and caste-specific expression differing for many genes in the heatmaps (Fig 3) and networks (Fig 4). For example, patterns of gene expression in the correlation networks (Fig 4) were consistent with interactions between the pre-optic area/arcuate nucleus in the context of GnRH regulation by KNDy neurons seen in other species [28, 34–35, 90] and appeared to be enhanced in females as compared to males. Specifically, in the arcuate nucleus, *Gpr147* was a larger node in females than in males; this brain region expresses the Gpr147 ligand, RFRP-3, at higher levels in subordinates than breeders [75] and could be reflecting greater reproductive suppression in females. In both sexes, *Kiss1* was also highly correlated with *Pdyn/Kor* in the arcuate nucleus, inferring possible up-regulation of these ligands relative to *Kiss1* to maintain suppression. While KNDy neurons have not been phenotyped in naked mole-rats, this pattern is consistent with other species, in which dynorphin signalling inhibits GnRH release [39–40, 91–92]. In males, but not females, there was a triadic relationship between *Tac3*, *Tac3r* and *Crhr1* in the arcuate nucleus, with *Tac3r* also being highly correlated to *Crhr2* and *Nr3c1;* in females, only *Tac3r* and *Nr3c1* were correlated. This suggests possible increased importance for *Tac3*/*Tac3r* in linking the HPA and HPG axes in males as compared to females, as has been suggested previously by Grachev et al. [41]. Sex differences in gene expression extend beyond the arcuate nucleus. There was an increased importance of gonadal gene expression, specifically the *Nr3c1* node, in the male network, whereas females had more connections between hippocampus (both dorsal and ventral) and other regions. Sex differences were also observed in the paraventricular nucleus and nucleus accumbens, discussed in more detail below. Collectively, these sex differences could indicate the neuroendocrine signals and brain regions regulating pubertal delay function in a sex-specific manner.

### A role for the nucleus accumbens in pubertal onset?

The nucleus accumbens likely plays an important role in reproductive maturation and/or social status transitions in naked mole-rats. We have previously reported an interaction between sex and status for oxytocin receptor binding in this region with breeding males tending to have higher receptor density than breeding females [89]. Kisspeptin-immunoreactive processes and CRHR1 receptor binding are present in both sexes [74, 84] though there is little to no androgen receptor immunoreactivity in either sex [88]. Here, we report that males had greater *Kiss1*, *Npvf*, *Gpr147* and *Tac3r* mRNA expression than females and that these sex differences often appeared larger in subordinates than in breeders (Fig 5). Several other genes also followed this pattern, but failed to reach statistical significance (see S1 Table).

Although the nucleus accumbens is typically studied for its role in the mesocorticolimbic circuitry associated with pleasure, reward and motivation [93–98], a smaller number of studies have investigated the nucleus accumbens in peri-puberty. In humans, nucleus accumbens volume decreases across the Tanner stages, although males exhibit a transient volumetric increase [99] and nucleus accumbens responsiveness to reward differs between adolescents and adults [100]. Alterations to the nucleus accumbens in pre- and peri-pubertal female prairie voles and male rats disrupts social processing, eliminating or reducing behaviors such as social attachment, alloparenting and social novelty seeking [101–102]. Thus, sex-specific changes in gene expression in the nucleus accumbens during naked mole-rat reproductive and social transitions could be associated with shifts in valence of psychosocial stimuli. Given the increased aggression directed towards subordinate females [103–105], the relatively lower nucleus accumbens gene expression in subordinate females might be equivalent to adolescents in whom accumbens hypoactivation and depression are associated with early life stress [106]. However, because gene expression levels are largely comparable between subordinate and breeding females, we propose the sex differences in gene expression in the nucleus accumbens stem from male hyperactivation, and not female hypoactivation, due to increased stress responsiveness in males.

### Crosstalk between HPG and HPA axes

In human and rat studies, adolescent and adult males show greater HPA responsiveness to (social) stress than females, with this male “stress hyperactivation” being more prominent in pre-pubertal male rats as compared to adult male rats [107–108]. The paraventricular nucleus is imperative to establishing social status effects due to its widely-explored associations with both stress [101–104] and socio-sexual behaviours [109–112]. In this study, we identified various sex-by-status interaction effects in the paraventricular/dorsomedial nuclei at the mRNA transcript level. The paraventricular nucleus is one possible region at the crossroads between the HPA and HPG axes in subordinate naked mole-rats, reacting to stress-related stimuli to delay pubertal timing. Both the paraventricular and dorsomedial nuclei of the hypothalamus express RFRP-3 in subordinates of both sexes [75]. At the mRNA transcript level, subordinate males have increased levels of the RFRP-3 receptor *Gpr147* relative to females, suggesting that GnRH suppression in subordinate males could originate from signalling from the paraventricular nucleus. Interestingly, a sex difference favoring males was observed in breeders (with males having higher expression of *Tac*3*r* and *Nr3c1*) whereas the sex difference was reversed in OS animals, with OS females having higher expression of *Tac*3*r* and *Nr3c1* relative to males. This reversal could be associated with peri-pubertal exposure to stress and changes in stress responsiveness [113–115]. Peri-pubertal stress results in *Crhr1-*mediated social deficits (Wister Han rats) [113] and increased responsiveness through corticotropin releasing hormone (mice) [116–117] or corticosterone (Fischer rats) [118].

Similar to the paraventricular nucleus, the present data indicate the arcuate nucleus integrates HPA axis signalling with the HPG axis through putative KNDy neuron regulation. As with the paraventricular nucleus, the arcuate nucleus expresses more RFRP-3 in subordinates compared to breeders [75] and we report here that subordinates have higher *Crhr2* as compared to breeders in this region. Ralph et al. [52] hypothesized that the HPA axis inhibits HPG signalling and thus reproduction via the KNDy population in the arcuate nucleus. Stress signals are potentially acting through *Tac3/Tac3r* in the arcuate nucleus, which interestingly is the node with the highest degree in the networks for both sexes (Fig 4). Grachev et al. [54] showed that corticotropin releasing hormone is communicating an inhibitory signal to *Tac3* in the arcuate nucleus to suppress the LH surge in female rats; however, this effect only occurs when animals are exposed to acute stress via lipopolysaccharide. We suggest that social stress might be triggering a similar mechanism (i.e., corticotropin releasing hormone and neurokinin B in the arcuate nucleus) to ultimately suppress HPG function in subordinate naked mole-rats.

The relationship between stress and social/reproductive status in naked mole-rats is not simple. Subordinates can but do not always have higher levels of cortisol than do breeders [119–121] and animals show evidence of reproductive activation in the presence of increased circulating cortisol (present data, [70–71]). In the bluehead wrasse (*Thalassoma bifasciatum*), the release from stress hypothesis suggests that a decrease in baseline HPI axis (equivalent to mammalian HPA axis) activity following removal of social stress allows for the initiation of female to male sex change [122]. Given the importance of social ascent in the life history of fish and putatively naked mole-rats, a role for the HPI/HPA axis is consistent with other life history transitions such as metamorphosis or smoltification [45]. In the bluebanded goby (*Lythyrpnus dalli*), social hierarchies led by a dominant male and female show the dominant female has elevated cortisol relative to the dominant male, while subordinates have intermediate cortisol levels relative to dominants [123]. In *Astatotilapia burtoni*, which also has socially-mediated reproductive suppression, subordinate animals released from suppression have elevated corticotropin-releasing hormone and its type 1 receptor within 15 minutes in both the pre-optic area and pituitary [124]. Furthermore, modifications to the glucocorticoid receptors appear to compensate for elevated cortisol levels in *A*. *burtoni* subordinates [125]. The present data suggest that similar status-specific HPA signaling occurs in naked mole-rats, including alterations in gene expression triggered by release from suppression. Another intriguing similarity between the *A*. *burtoni* model of reproductive suppression and the naked mole-rat is the identification of the nucleus accumbens for processing of related social stimuli. Dopaminergic control of GnRH1 neurons has been identified in the ventral telencephalon, a proposed nucleus accumbens homologue, which is known to facilitate social cognition in *A*. *burtoni* subordinates [126–127]. Together, these data from two diverse vertebrate species (the teleost *A*. *burtoni* and the mammalian naked mole-rat) suggest a conserved importance for the nucleus accumbens in socially-mediated reproductive suppression.

### Opposite-sex paired animals provide insight into reproductive and social transitions

The OS group was originally included as a transition group to tease apart reproduction and social status differences at the molecular level. These animals were released from the suppressive cues of their natal colony but had not yet produced their own pups. Indeed, we identified expression differences between groups where a subset of the genes quantified matched either subordinate or breeder expression profiles. Interestingly, we also identified a large number of genes whose expression pattern matches neither subordinate nor breeding animals (Fig 3). These genes might represent a unique pattern of expression required for pubertal transitions or, more likely in our opinion, a response to relative social isolation. Removal from the colony and social pairing increases circulating cortisol, which can persist for at least one month (present data; 76) and is coincident with reproductive activation [70]. Thus, relative social isolation might trigger a stress response independent of pubertal onset. Studying additional time points during the transition, in addition to varying the degree of social isolation (e.g., removing and co-housing multiple animals simultaneously), will help shed light on this issue.

### Conclusions and future directions

To our knowledge, this work is the first investigation of how reproduction- and stress-related genes are expressed upstream of GnRH in naked mole-rats, providing insight into mechanisms of socially-controlled pubertal onset across brain regions. We suggest that this process occurs in a sexually differentiated manner and identify gene candidates for future directed hypothesis testing. Indeed, several genes (e.g. *Tac3*, *Crhr2*, *Nr3c1*) warrant future focused investigation for understanding how neuroendocrine circuits can indefinitely suppress puberty in this species. Future studies should examine puberty onset in naked mole-rats using genome-wide techniques to identify novel genes and pathways involved in socially-controlled reproductive suppression and subsequent activation. Building on the nodes identified herein, we can begin to work outwards to connect the pathways and develop sex-specific pubertal gene regulatory networks.

## Supporting information

S1 Datasheet Summary of all normalized data. Summary of all data used in analyses: individual gene qPCR relative expression, hormonal assays, weight, age, experimental group and sex. *OS = opposite-sex paired animal*, *PVN = paraventricular and dorsomedial nucleus*, *NAcc = nucleus accumbens*, *Arc = arcuate nucleus/median eminence*, *POA = pre-optic area*, *DHipp = dorsal hypothalamus*, *VHipp = ventral hypothalamus*, *MeA = medial amygdala*.
(XLSX)

S1 Table Summary of all sex-by-group ANOVA results. Statistical significance is considered based on the critical alpha 0.05 (p<0.05). The experimental group exhibiting greater expression is specified in brackets following the factor for significant results only. An asterisk denotes a region in which the gene was not analyzed. *SxG = Sex by group*, *B = BRE*, *S = SUB*, *OS = OS*, *M = Male*, *F = Female*, *PVN = paraventricular and dorsomedial nucleus*, *NAcc = nucleus accumbens*, *Arc = arcuate nucleus/median eminence*, *POA = pre-optic area*, *DHipp = dorsal hypothalamus*, *VHipp = ventral hypothalamus*, *MeA = medial amygdala*.
(PDF)
