# Supplementary material for: Sex- and brain region-specific patterns of gene expression associated with socially-mediated puberty in a eusocial mammal
Source: PLoS One. 2018 Feb 23;13(2):e0193417. doi: 10.1371/journal.pone.0193417 (PMC5825099; doi:10.1371/journal.pone.0193417)
Supplement: S1 Table — Statistical significance is considered based on the critical alpha 0.05 (p<0.05). The experimental group exhibiting greater expression is specified in brackets following the factor for significant results only. An asterisk denotes a region in which the gene was not analyzed. SxG = Sex by group, B = BRE, S = SUB, OS = OS, M = Male, F = Female, PVN = paraventricular and dorsomedial nucleus, NAcc = nucleus accumbens, Arc = arcuate nucleus/median eminence, POA = pre-optic area, DHipp = dorsal hypothalamus, VHipp = ventral hypothalamus, MeA = medial amygdala. (PDF) [file pone.0193417.s002.pdf]

S1 Table. Summary of all sex-by-group ANOVA results. Statistical significance is considered based on the critical alpha 0.05 (p<0.05). The experimental group exhibiting greater expression is specified in brackets following the factor for significant results only. An asterisk denotes a region in which the gene was not analyzed. *SxG=Sex by group, B=BRE, S=SUB, OS=OS, M=Male, F=Female, PVN=paraventricular and dorsomedial nucleus, NAcc=nucleus accumbens, Arc=arcuate nucleus/median eminence, POA=pre-optic area, DHipp=dorsal hypothalamus, VHipp=ventral hypothalamus, MeA=medial amygdala*

Region	PVN	NAcc	Arc	POA	MeA	DHipp	VHipp	Gonads
Npvf	SxG: F(2,37)=0.242, p=0.786<br>Sex: F(1,37)=0.255, p=0.617<br>Group: F(2,37)=0.075, p=0.928	SxG: F(2,36)=1.94, p=0.158<br>**Sex (M): F(1,36)=4.96, p=0.032**<br>Group: F(2,36)=0.837, p=0.441	SxG: F(2,37)=0.476, p=0.625<br>Sex: F(1,37)=0.001, p=0.977<br>Group: F(2,37)=0.548, p=0.582	SxG: F(2,36)=2.48, p=0.098<br>Sex: F(1,36)=2.39, p=0.131<br>Group: F(2,36)=1.00, p=0.377	*	*	*	SxG: F(2,38)=0.222, p=0.802<br>Sex: F(1,38)=3.32, p=0.076<br>Group: F(2,38)=1.08, p=0.350
Gpr147	SxG: F(2,32)=0.510, p=0.605<br>**Sex (M): F(1,32)=4.24, p=0.048**<br>Group: F(2,32)=0.106, p=0.899	**SxG (BF>SF, SM>SF): F(2,34)=3.37, p=0.046**<br>Sex: F(1,34)=1.14, p=0.294<br>Group: F(2,34)=0.445, p=0.645	SxG: F(2,30)=1.65, p=0.210<br>Sex: F(1,30)=1.56, p=0.216<br>Group: F(2,30)=0.464, p=0.633	SxG: F(2,37)=1.55, p=0.225<br>Sex: F(1,37)=0.272, p=0.605<br>Group: F(2,37)=1.31, p=0.283	*	*	*	SxG: F(2,35)=2.40, p=0.106<br>Sex: F(1,35)=0.965, p=0.333<br>Group: F(2,35)=0.787, p=0.463
Kiss1	SxG: F(2,34)=1.20, p=0.314<br>Sex: F(1,34)=1.51, p=0.227<br>Group: F(2,34)=0.585, p=0.563	SxG: F(2,36)=0.211, p=0.811<br>Sex: F(1,36)=4.04, p=0.052<br>Group: F(2,36)=0.105, p=0.901	SxG: F(2,35)=1.90, p=0.165<br>Sex: F(1,35)=1.12, p=0.297<br>Group: F(2,35)=0.047, p=0.954	SxG: F(2,35)=1.48, p=0.243<br>Sex: F(1,35)=0.839, p=0.366<br>Group: F(2,35)=0.172, p=0.843	*	*	*	*
Kiss1r	SxG: F(2,37)=1.24, p=0.300	SxG: F(2,38)=0.332, p=0.719	SxG: F(2,35)=1.90, p=0.165	SxG: F(2,35)=1.03, p=0.367	*	*	*	*

	Sex: F(1,37)=0.198, p=0.659<br>Group: F(2,37)=0.507, p=0.606	Sex: F(1,38)=3.13, p=0.579<br>Group: F(2,38)=2.75, p=0.077	Sex: F(1,35)=1.12, p=0.297<br>Group: F(2,35)=0.047, p=0.954	Sex: F(1,35)=3.48, p=0.071<br>Group: F(2,35)=2.44, p=0.102				
Tac3	SxG: F(2,37)=1.93, p=0.160<br>Sex: F(1,37)=0.00, p=0.99<br>Group: F(2,37)=0.356, p=0.703	**SxG (SM): F(2,34)=3.41, p=0.045**<br>Sex: F(1,34)=0.031, p=0.862<br>Group: F(2,34)=0.198, p=0.821	SxG: F(2,36)=1.15, p=0.327<br>Sex: F(1,36)=0.849, p=0.363<br>Group: F(2,36)=1.735, p=0.191	SxG: F(2,36)=0.205, p=0.815<br>Sex: F(1,36)=0.001, p=0.976<br>Group: F(2,36)=0.979, p=0.385	*	*	*	*
Tac3r	**SxG (BM>SM/OSM/BF): F(2,37)=5.99, p=0.005)**<br>Sex: F(1,37)=0.00, p=0.996<br>Group: F(2,37)=1.27, p=0.292	SxG: F(2,37)=2.42, p=0.102<br>**Sex (M): F(1,37)=4.10, p=0.050**<br>Group: F(2,37)=1.64, p=0.207	SxG: F(2,35)=2.25, p=0.120<br>Sex: F(1,35)=1.12, p=0.300<br>Grouo: F(2,35)=0.582, p=0.564	SxG: F(2,34)=0.903, p=0.415<br>Sex: F(1,34)=0.639, p=0.430<br>Group: F(2,34)=1.29, p=0.289	*	*	*	*
Pdyn	SxG: F(2,36)=1.49, p=0.239<br>Sex: F(1,36)=0.646, p=0.427<br>Group: F(2,36)=0.328, p=0.723	SxG: F(2,38)=0.094, p=0.910<br>Sex: F(1,38)=1.83, p=0.184<br>Group: F(2,38)=0.144, p=0.866	SxG: F(2,34)=1.43, p=0.254<br>Sex: F(1,34)=1.42, p=0.242<br>Group: F(2,34)=0.210, p=0.812	SxG: F(2,33)=0.777, p=0.468<br>Sex: (1,33)=0.533, p=0.470<br>Group: F(2,33)=1.97, p=0.155	*	*	*	*

Kor	SxG: F(2,33)=1.66, p=0.206 Sex: F(1,33)=4.00, p=0.054 Group: F(2,33)=1.54, p=0.230	SxG: F(2,36)=1.74, p=0.189 Sex: F(1,36)=0.967, p=0.332 Group: F(2,36)=0.967, p=0.390	SxG: F(2,36)=1.20, p=0.314 Sex: F(1,36)=0.032, p=0.859 Group: F(2,36)=0.278, p=0.759	SxG: F(2,34)=1.71, p=0.196 Sex: F(1,34)=1.43, p=0.239 Group: F(2,34)=2.07, p=0.142	*	*	*	*
Nr3c1	**SxG (BM>BF): F(2,35)=7.18, p=0.002** Sex: F(1,35)=0.338, p=0.565 Group: F(2,35)=2.84, p=0.072	SxG: F(2,37)=0.755, p=0.477 Sex: F(1,37)=0.081, p=0.777 Group: F(2,37)=2.01, p=0.148	SxG: F(2,36)=1.64, p=0.209 Sex: F(1,36)=0.834, p=0.367 Group: F(2,36)=0.110, p=0.896	SxG: F(2,35)=1.71, p=0.195 Sex: F(1,35)=0.225, p=0.639 Group: F(2,35)=0.082, p=0.921	SxG: F(2,36)=1.122, p=0.337 Sex: F(1,36)=1.036, p=0.316 Group: F(2,36)=0.145, p=0.866	SxG: F(2,37)=0.258, p=0.774 Sex: F(1,37)=0.410, p=0.526, **Group (S>OS): F(2,37)=4.02, p=0.026**	SxG: F(2,36)=0.758, p=0.476 Sex: F(1,36)=0.276, p=0.603 Group: F(2,36)=1.99, p=0.151	SxG: F(2,36)=3.22, p=0.052 Sex: F(1,36)=0.014, p=0.907 Group: F(2,36)=0.418, p=0.661
Crhr1	SxG: F(2,36)=0.622, p=0.542 Sex: F(1,36)=1.53, p=0.224 Group: F(2,36)=3.02, p=0.061	SxG: F(2,37)=0.589, p=0.560, Sex: F(1,37)=2.41, p=0.129 Group: F(2,37)=0.653, p=0.527	SxG: F(2,37)=0.612, p=0.548 Sex: F(1,37)=0.024, p=0.877 Group: F(2,37)=1.43, p=0.253	SxG: F(2,35)=1.24, p=0.301 Sex: F(1,35)=0.903, p=0.348 Group: F(2,35)=0.764, p=0.473	SxG: F(2,37)=0.134, p=0.875 Sex: F(1,37)=1.95, p=0.171 Group: F(2,37)=1.828, p=0.175	SxG: F(2,34)=1.03, p=0.368 Sex: F(1,34)=2.56, p=0.119 Group: F(2,34)=0.198, p=0.821	SxG: F(2,37)=0.864, p=0.430 Sex: F(1,37)=1.63, p=0.210 Group: F(2,37)=0.736, p=0.486	*
Crhr2	**SxG (BM): F(2,37)=0.622, p=0.042** Sex: F(1,37)=1.53, p=0.162 **Group (B/OS): F(2,37)=3.02, p=0.027**	SxG: F(2,36)=1.62, p=0.212 Sex: F(1,36)=2.90, p=0.098 **Group (OS): F(2,36)=3.68, p=0.035**	SxG: F(2,34)=1.58, p=0.221 Sex: F(1,34)=0.049, p=0.827 **Group (S>B): F(2,34)=3.53, p=0.040**	SxG: F(2,37)=0.558, p=0.577 Sex: F(1,37)=1.93, p=0.173 Group: F(2,37)=1.58, p=0.220	SxG: F(2,36)=0.358, p=0.702 Sex: F(1,36)=3.32, p=0.077 **Group (S): F(2,36)=4.20, p=0.023**	SxG: F(2,36)=1.52, p=0.233 Sex: F(1,36)=0.799, p=0.377 Group: F(2,36)=0.656, p=0.525	**SxG (OSM>OSF/BM/SM): F(2,34)=4.56, p=0.018** Sex: F(1,34)=0.030, p=0.863 Group: F(2,34)=0.172, p=0.843	*
